# The Effect of RADA16-I and CDNF on Neurogenesis and Neuroprotection in Brain Ischemia-Reperfusion Injury

**DOI:** 10.3390/ijms23031436

**Published:** 2022-01-27

**Authors:** Xingyu Liu, Haiyuan Ren, Ai Peng, Haoyang Cheng, Jiahao Chen, Xue Xia, Ting Liu, Xiaojing Wang

**Affiliations:** Department of Cell Biology, School of Basic Medical Sciences, Cheeloo College of Medicine, Shandong University, Jinan 250012, China; 201714954@mail.sdu.edu.cn (X.L.); ren15993562826@163.com (H.R.); pengai1007@163.com (A.P.); 201800412079@mail.sdu.edu.cn (H.C.); cjh578327358@126.com (J.C.); xx1556169939@163.com (X.X.); liuting@sdu.edu.cn (T.L.)

**Keywords:** RADA16-I, CDNF, NSCs, ischemia-reperfusion

## Abstract

Scaffold materials, neurotrophic factors, and seed cells are three elements of neural tissue engineering. As well-known self-assembling peptide-based hydrogels, RADA16-I and modified peptides are attractive matrices for neural tissue engineering. In addition to its neuroprotective effects, cerebral dopamine neurotrophic factor (CDNF) has been reported to promote the proliferation, migration, and differentiation of neural stem cells (NSCs). However, the role of RADA16-I combined with CDNF on NSCs remains unknown. First, the effect of RADA16-I hydrogel and CDNF on the proliferation and differentiation of cultured NSCs was investigated. Next, RADA16-I hydrogel and CDNF were microinjected into the lateral ventricle (LV) of middle cerebral artery occlusion (MCAO) rats to activate endogenous NSCs. CDNF promoted the proliferation of NSCs, while RADA16-I induced the neural differentiation of NSCs in vitro. Importantly, both RADA16-I and CDNF promoted the proliferation, migration, and differentiation of endogenous NSCs by activating the ERK1/2 and STAT3 pathways, and CDNF exerted an obvious neuroprotective effect on brain ischemia-reperfusion injury. These findings provide new information regarding the application of the scaffold material RADA16-I hydrogel and the neurotrophic factor CDNF in neural tissue engineering and suggest that RADA16-I hydrogel and CDNF microinjection may represent a novel therapeutic strategy for the treatment of stroke.

## 1. Introduction

Scaffold materials, neurotrophic factors, and seed cells, the three elements of neural tissue engineering, play an important role in the field of regenerative medicine of the nervous system [1,2]. In neural tissue engineering, numerous natural and synthetic materials have been investigated for the fabrication of scaffolds. Among these materials, hydrogels, which are three-dimensional (3D) networks formed by hydrophilic polymers containing up to 90% (*w*/*w*) water, have attracted much attention due to their biocompatibility, biodegradability, interactivity, porosity and lack of swelling [3]. They display very attractive properties for future clinical applications in neuroregeneration. RADA16-I, a well-known self-assembling peptide-based hydrogel, can organize into nanofiber scaffolds through a self-assembly process [4]. The effects of the RADA16-I hydrogel and its modification with laminin have been investigated using human NPCs. The modified peptide induced cell differentiation with the highest dopaminergic maturation of neurons [5]. Human ES-NPCs growing with RADA16-I compared to the laminin-coated surface were characterized. The results showed that RADA16-I increased cell survival and stimulated the differentiation and maturation of the cells. Neurite outgrowth was greater with the 0.25% RADA16-I hydrogel than with the laminin-coated surface [6]. RADA16-I and sterile saline were injected into the brain injury sites in a rat TBI model. The cavity injected with self-assembling peptides was significantly reduced in size and integrated with no obvious gaps. However, RADA16-I did not promote neural lineage cell migration or oligodendrocyte migration [7]. RADA16-I or RADA16-IKVAV was injected into rats after traumatic brain injury. The results showed that both hydrogels supported survival and promoted proliferation of the encapsulated cells. However, IKVAV-modified hydrogels promoted proliferation more prominently [8]. Human iPS-NPCs were encapsulated into RADA16-I microspheres and then injected into the mouse striata. Neurite outgrowth was promoted, and cell survival was enhanced [9]. Given these findings, RADA16-I and modified peptides are attractive matrices for neural tissue engineering.

Neurotrophic factors (NTFs), as an important group of secreted proteins, can regulate neural system development and have neuroprotective roles in the survival or regeneration of neurons in the injured brain and neurodegenerative diseases. Many families of NTFs have been studied, including nerve growth factor (NGF), brain-derived neurotrophic factor (BDNF), neurotrophin-3 (NT-3) and NT-4 and glial cell line-derived neurotrophic factor (GDNF) [10]. To date, although these NTFs have many beneficial effects on neurons, the outcomes of clinical trials on neurodegenerative diseases have not been very successful [10,11,12]. CDNF, a member of the novel and evolutionarily conserved CDNF/MANF family, has shown neurotrophic activities in dopaminergic neurons [13,14,15]. Published data from our group demonstrated the neuroprotective effects of CDNF on cerebral ischemia and the oxygen-glucose deprivation (OGD) cell model, which may occur through ER stress pathways [16]. Human CDNF exhibits 59% amino acid identity with human mesencephalic astrocyte-derived neurotrophic factor (MANF), another member of the CDNF/MANF family. MANF has been reported to promote the differentiation and migration of neural progenitor cells in an OGD model and a rat model of cortical stroke [17]. CDNF enhances the proliferation and migration of NSCs toward the lesioned striatum in Parkinsonian rats [18]. CDNF was able to reverse the adverse proliferation, differentiation, and apoptotic effects that normally affect NSCs in a hypoxic environment [19]. There are no reported data on CDNF on NSCs in a cerebral ischemia model.

As stem cells in the nervous system, NSCs that can differentiate into neurons, astrocytes and oligodendrocytes are ideal candidates as seed cells for neural tissue engineering and regenerative medicine [20,21]. In the adult mammalian brain, NSCs are primarily located in the subventricular zone (SVZ) of the LV and the subgranular zone (SGZ) in the hippocampal dentate gyrus (DG) [22]. Due to the decreased viability of cell transplantation, endogenous NSCs are a good resource to utilize. Ischemic injury drastically increases neurogenesis in the rodent SVZ, and NSCs in the SVZ can generate many neuroblasts and migrate to the ischemic penumbra, improving neurological functional recovery [23,24]. However, self-repair from endogenous NSCs is very limited due to a nonpermissive environment for migration and neuronal differentiation [25,26].

In this study, we examined the effect of RADA16-I hydrogel combined with CDNF on NSCs in vitro and then microinjected them into the LV to activate endogenous NSCs in the SVZ. Neurogenesis and neuroprotection were explored in a rat MCAO model. The results demonstrated that CDNF promoted the proliferation of NSCs and that RADA16-I induced the neuronal differentiation of NSCs in vitro. Most importantly, the in vivo data demonstrated that both RADA16-I and CDNF promoted the proliferation of NSCs and neuroblasts in the SVZ and the migration of neuroblasts to the ischemic penumbra by activating the ERK1/2 and STAT3 pathways. Compared to RADA16-I, CDNF exerted a significant neuroprotective effect on brain ischemia-reperfusion injury. This finding indicates the possibility of activating endogenous NSCs for brain injury repair using RADA16-I hydrogel combined with CDNF.

## 2. Results

### 2.1. CDNF, but Not RADA16-I, Promotes the Proliferation of Cultured NSCs

**Figure 1:**
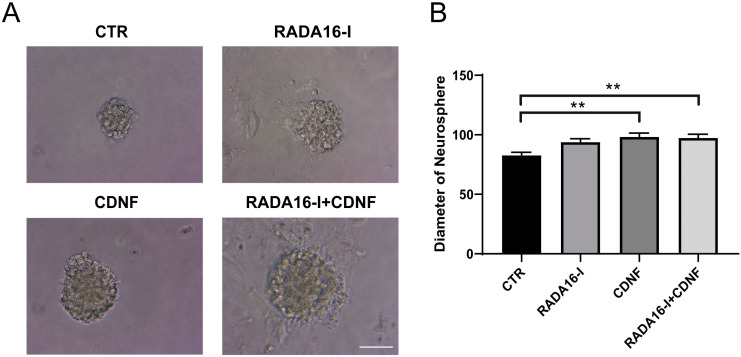
CDNF promoted the proliferation of cultured NSCs. (**A**) Light-microscopic images of NSCs cultured in neurosphere on the fifth day. Scale bar = 25 μm. (**B**) The diameter of neurospheres in each group was quantified. Values represent the mean ± SEM, ** *p* < 0.01 according to ANOVA followed by LSD post hoc analysis.

For the cultured NSCs, each well of 24-well dishes was precoated with 50 µL 1% RADA16-I 24 h before cell plating in the RADA16-I and RADA16-I+CDNF groups. The third day after cell plating, CDNF (50 ng/mL) was added to the culture medium in the CDNF and RADA16-I + CDNF groups. On the fifth day, the neurospheres in each group were imaged. Under a light microscope, larger neurospheres were observed in the RADA16-I, CDNF and RADA16-I + CDNF groups than in the control group (Figure 1A). Then, the diameter of the neurospheres was measured. The results showed that the diameter of neurospheres was significantly different among these groups (F (3, 335) = 4.697; *p* < 0.01, Figure 1B). Post hoc analysis indicated this CDNF treatment was significantly different from that of the no treatment control group (*p* = 0.006). Such a large diameter of neurospheres suggests that the neural stem cells in neurospheres strongly proliferated. Significant increases in the diameter of neurospheres were observed in the RADA16-I + CDNF treatment group compared to the control group. No statistically significant differences were obtained between only RADA16-I treatment and the control (*p* > 0.05). These results indicate that CDNF, not RADA16-I, promotes the proliferation of neural stem cells in vitro.

### 2.2. RADA16-I, but Not CDNF, Promotes the Neural Differentiation of Cultured NSCs

Withdrawing the growth factors, fetal bovine serum (1%) was added to the culture medium of neural stem cells to induce differentiation. Seven days after differentiation, markers of GFAP and Tuj1 were used to identify glial cells and neural cells, respectively, that were derived from the NSCs.

**Figure 2:**
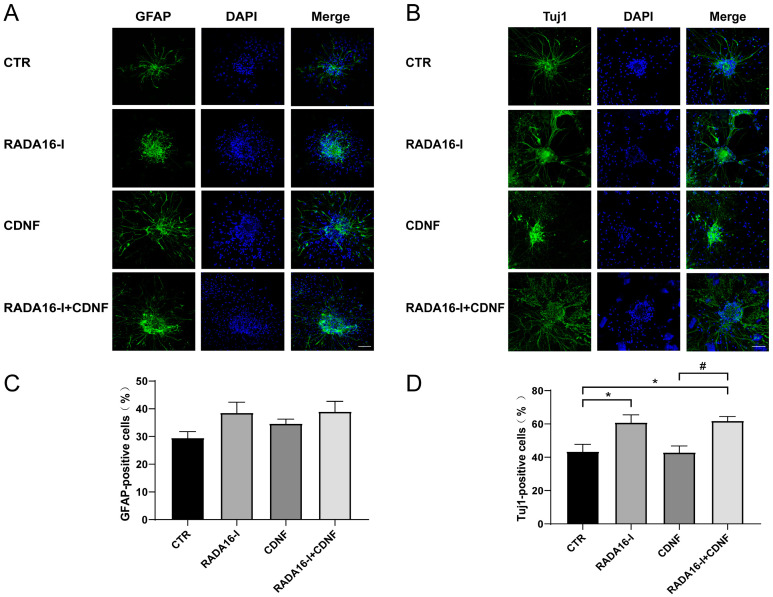
The effect of RADA16-I and CDNF on the differentiation of cultured NSCs. (**A**) GFAP-positive cells (green) were identified via immunohistochemical staining. DAPI (blue) was used to label the nuclei. (**B**) Tuj1-positive cells (green) were identified via immunohistochemical staining. DAPI (blue) was used to label the nuclei. (**C**) Quantitative analysis of the number of GFAP-positive cells. (**D**) Quantitative analysis of the number of Tuj1-positive cells. * *p* < 0.05 indicates statistical significance between treatment group and CTR group; # *p* < 0.05 indicates statistical significance between CDNF group and RADA16-I + CDNF group. Scale bar = 45 μm.

Immunocytofluorescence staining produced an overall statistically significant increase in the ratio of neural differentiation (F (3, 19) = 7.419; *p* < 0.01, Figure 2A,C). Post hoc analysis indicated that treatment with RADA16-I and RADA16-I + CDNF significantly increased the ratio of Tuj1-positive cells (*p* < 0.05). Treatment with CDNF did not exert a significant difference in neural differentiation compared to the control group (*p* < 0.05). However, there was no significant difference in glial differentiation between these treatments (F (3, 19) = 2.247; *p* = 0.116, Figure 2B,D). The ratio of GFAP-positive cells was not significantly different between these groups (*p* > 0.05). The staining results demonstrated that RADA16-I, but not CDNF, induced neural stem cells to differentiate into neural cells rather than glial cells.

### 2.3. RADA16-I and CDNF Promote the Proliferation of NSCs and Neuroblasts in the SVZ of Ischemia/Reperfusion Rats

**Figure 3:**
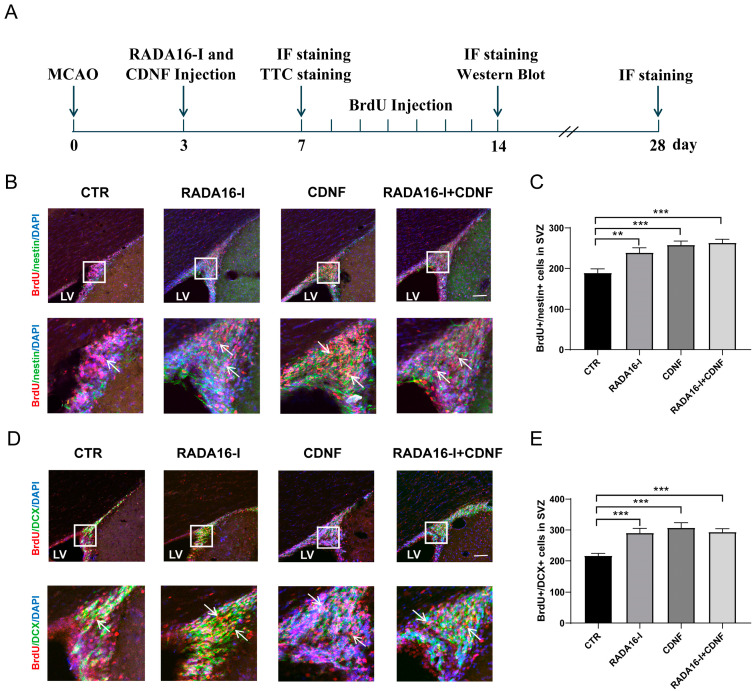
RADA16-I and CDNF promoted the proliferation of NSCs and neuroblasts in the SVZ of MCAO rats. (**A**) Experimental scheme of treatment with RADA16-I and CDNF in the MCAO rats. (**B**) RADA16-I and CDNF significantly increased the number of BrdU (red)/nestin (green)-positive cells in the SVZ two weeks after MCAO. DAPI (blue) was used to label the nuclei. The insets show photomicrographs of the SVZ at higher magnification. Arrows point to the BrdU/nestin-positive cells. (**C**) Quantitative analysis of BrdU/nestin-positive cells in the SVZ. (**D**) RADA16-I and CDNF significantly increased the number of BrdU (red)/DCX (green)-positive cells in the SVZ. DAPI (blue) was used to label the nuclei. The insets show photomicrographs of the SVZ at higher magnification. Arrows point to the BrdU/DCX-positive cells. (**E**) Quantitative analysis of BrdU/DCX-positive cells in the SVZ. Every sixth section of whole SVZ in each rat was collected for staining. *N* = 5/group. ** *p* < 0.01, *** *p* < 0.001 according to ANOVA followed by LSD post hoc analysis. Scale bar = 45 μm. LV, lateral ventricle.

To determine the effect on neurogenesis and neuroprotection in vivo, RADA16-I and CDNF were injected into the right lateral ventricle using a stereotaxic apparatus in the MCAO rat model. BrdU was injected seven days from days 7 to 14 (Figure 3A). BrdU staining labels proliferating cells. Nestin, a member of the intermediate filament protein family, is expressed by neuroepithelial precursor cells [27]. DCX is an immature neuronal marker that can label neuroblasts. Double labeled immunohistochemistry of BrdU/nestin and BrdU/DCX in brain slices labeled neural stem cells and neuroblasts in the SVZ of MCAO rats. After administration for fourteen days, the brains were removed and sectioned after the rats were sacrificed.

Immunofluorescence staining of brain slices revealed that BrdU/nestin-positive cells were significantly increased in the SVZ area in these treated groups (F (3, 93) = 13.41; *p* < 0.0001, Figure 3B,C). Post hoc analysis indicated that a greater number of BrdU/nestin-positive cells was observed in the SVZ of the RADA16-I treatment group than in the control group (*p* = 0.005). Both the CDNF alone treatment and the RADA16-I + CDNF treatment groups exhibited significantly more BrdU/nestin-positive cells than the control group (*p* < 0.0001). These results indicate that treatment with RADA16-I, CDNF alone, or RADA16-I + CDNF promotes the proliferation of NSCs in the SVZ of MCAO rats.

Next, we aimed to determine whether the proliferation of neuroblasts within the SVZ was affected by treatment. An overall statistically significant difference was observed for the number of BrdU/DCX-positive cells (F (3, 86) = 16.74; *p* < 0.0001, Figure 3D,E) among these groups. Post hoc analysis indicated that the three treatment groups displayed significantly more BrdU/DCX-positive cells within the SVZ than the control group (*p* < 0.0001). These results indicate that treatment with RADA16-I, CDNF alone, or RADA16-I + CDNF promotes the proliferation of neuroblasts in the SVZ of MCAO rats.

In order to verify the promoting effect for the proliferation of NSCs and neuroblasts, the effects of RADA16-I and CDNF on the cell death of newly divided cells need to be excluded. No significant difference was observed in the cell death of newly divided cells in SVZ among these groups (F (3, 23) = 3.342; *p* = 0.191, Figure S1A,B). The number of BrdU/cleaved caspase-3-positive cells were not significantly different between these groups (*p* > 0.05). These results indicate treatment with RADA16-I, CDNF alone, or RADA16-I + CDNF cannot suppress the cell death of newly divided cells in the SVZ of MCAO rats.

### 2.4. CDNF, but Not RADA16-I, Promotes the Migration of Neuroblasts in the SVZ into the Ischemic Penumbra Area of Ischemia/Reperfusion Rats

In the normal brain, neural stem cells within the SVZ proliferate and differentiate into neuroblasts that migrate to the olfactory bulb via the rostral migratory stream [28]. However, neuroblasts derived from the SVZ migrate toward the ischemic striatum and cortex and differentiate into neurons in the MCAO model [23,25,29,30,31,32,33]. Highly efficient migration of neuroblasts to the ischemic area is crucial for the cell replacement treatment of stroke.

**Figure 4:**
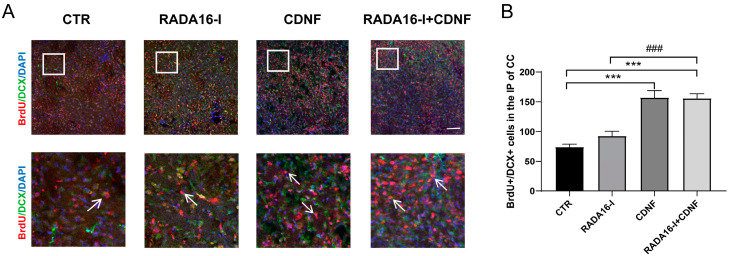
CDNF promoted the migration of neuroblasts in the SVZ into the ischemic penumbra area of MCAO rats. (**A**) BrdU (red)/DCX (green)-positive cells were identified via immunohistochemical staining. DAPI (blue) was used to label the nuclei. The insets show photomicrographs of the ischemic penumbra at higher magnification. Arrows point to the BrdU/DCX-positive cells. Scale bar = 45 μm. (**B**) Quantitative analysis of BrdU/DCX-positive cells in the ischemic penumbra. *N* = 5/group. *** *p* < 0.001 indicates statistical significance between treatment group and CTR group; ### *p* < 0.001 indicates statistical significance between RADA16-I group and RADA16-I + CDNF group.

Whether the increased neuroblasts within the SVZ can migrate into the ischemic prenumbra area remains unclear. To determine the effect of RADA16-I and CDNF on migration, we further examined BrdU/DCX-positive cells in the ischemic prenumbra area of the cerebral cortex. Double labeled immunohistochemistry showed that cell migration was significantly increased in the ischemic prenumbra area (F (3, 81) = 31.696; *p* < 0.0001, Figure 4A,B). Post hoc analysis indicated that treatment with CDNF and RADA16-I + CDNF significantly increased the number of BrdU/DCX-positive cells compared to that of the control group (*p* < 0.0001). Additionally, the RADA16-I + CDNF group displayed more BrdU/DCX-positive cells than the RADA16-I group (*p* < 0.0001). No significant difference was observed between the RADA16-I and control groups (*p* > 0.05). These findings demonstrate that CDNF, not RADA16-I, promotes the migration of neuroblasts from the SVZ to the ischemic penumbra region in ischemia/reperfusion rats.

### 2.5. RADA16-I and CDNF Promote Neural Differentiation in Ischemia/Reperfusion Rats

**Figure 5:**
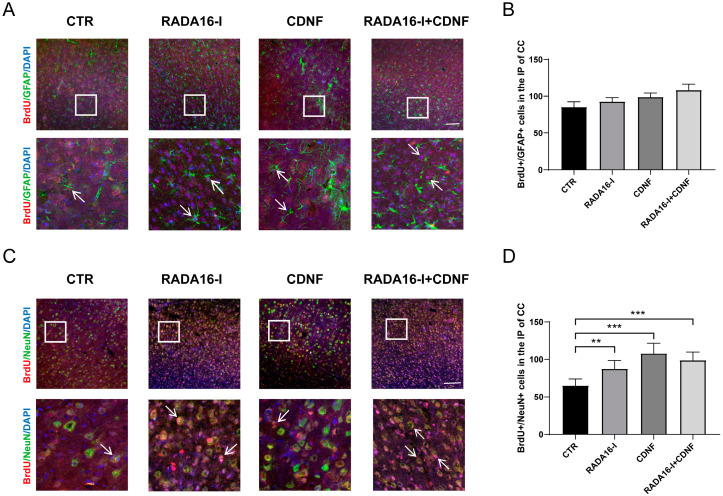
RADA16-I and CDNF promoted neural differentiation in MCAO rats. (**A**) BrdU (red)/GFAP (green)-positive cells were identified in the ischemic prenumbra four weeks after MCAO. DAPI (blue) was used to label the nuclei. The insets show photomicrographs of the ischemic penumbra at higher magnification. Arrows point to the BrdU/GFAP-positive cells. (**B**) Quantitative analysis of BrdU/GFAP-positive cells in the ischemic penumbra. (**C**) BrdU (red)/NeuN (green)-positive cells were identified in the ischemic prenumbra four weeks after MCAO. DAPI (blue) was used to label the nuclei. The insets show photomicrographs of the ischemic penumbra at higher magnification. Arrows point to the BrdU/NeuN-positive cells. (**D**) Quantitative analysis of BrdU/NeuN-positive cells in the ischemic penumbra. *N* = 5/group. ** *p* < 0.01, *** *p* < 0.001 according to ANOVA followed by LSD post hoc analysis. Scale bar = 45 μm.

To investigate the effect of RADA16-I and CDNF on the cell differentiation of NSCs in the ischemic prenumbra region of the cerebral cortex. Four weeks after MCAO, double labeled immunohistochemistry was performed to identify the cell differentiation of NSCs derived from the SVZ. BrdU/GFAP staining labeled new astrocytes, while BrdU/NeuN staining labeled new neurons. No significant difference was obtained in the number of BrdU/GFAP-positive cells ((F(3, 47) = 2.061; *p* = 0.1182); post hoc *p* > 0.05, Figure 5A,B) among these groups. However, significantly increased BrdU/NeuN-positive cells were observed in all the treatment groups compared to the control group ((F(3, 56) = 15.528; *p* < 0.001); post hoc *p* < 0.01, Figure 5C,D). No significant difference was observed among the treatment groups (*p* > 0.05). These results indicated that CDNF and RADA16-I promoted neural cell differentiation of the NSC-derived SVZ in the ischemic prenumbra area of the cerebral cortex.

### 2.6. Pathways Involved in the Neurogenesis of CDNF and RADA16-I in Ischemia/Reperfusion Rats

**Figure 6:**
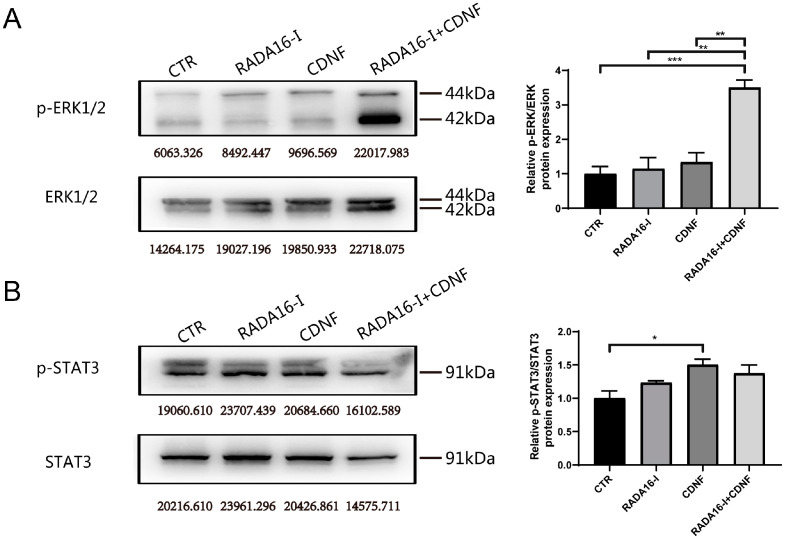
Pathways involved in the neurogenesis of CDNF and RADA16-I in MCAO rats. (**A**) Western blot analysis of *p*-ERK1/2 and ERK1/2. The protein levels of *p*-ERK1/2 were quantified relative to the level of ERK1/2. *N* = 4/group. (**B**) Levels of *p*-STAT3-Ser727 and STAT3 were determined with use of Western blotting. The protein levels of *p*-STAT3-Ser727 were quantified relative to the level of STAT3. *N* = 4/group. * *p* < 0.05, ** *p* < 0.01, *** *p* < 0.001 according to ANOVA followed by LSD post hoc analysis.

To deeply explore the molecular mechanisms involved in the neurogenesis of NSCs in the SVZ, immunoblotting was performed to identify the important pathways. ERK1/2 and STAT3 pathways were investigated. The results showed that expression levels of phosphorylated ERK1/2 (*p*-ERK1/2) were significantly increased in the RADA16-I + CDNF group compared to the other groups (F (3, 8) = 16.614; *p* < 0.001, post hoc *p* < 0.001, Figure 6A), and expression levels of Ser727 phosphorylated STAT3 (*p*-STAT3) were significantly elevated in the CDNF treatment groups compared to the control group (F (3, 12) = 5.233; *p* = 0.015, post hoc *p* = 0.016, Figure 6B). These results indicate that the ERK1/2 and STAT3 pathways may be involved in the neurogenesis promoted by CDNF and RADA16-I treatment.

### 2.7. CDNF, but Not RADA16-I, Has a Neuroprotective Effect on Ischemia/Reperfusion Rats

CDNF has been demonstrated to have a neuroprotective effect on ischemic injury in MCAO rats in published data from our lab [16]. The goal of this experiment was to determine whether RADA16-I has a neuroprotective effect or synergistic effect with CDNF in MCAO rats.

**Figure 7:**
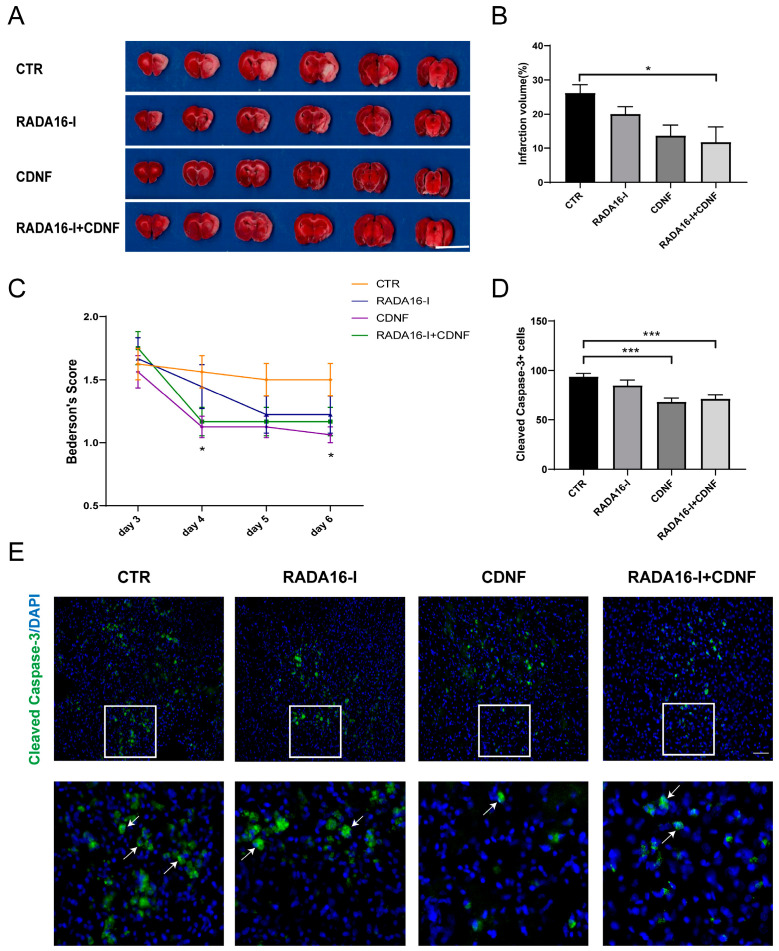
CDNF had a neuroprotective effect in brain ischemia-reperfusion injury. (**A**) TTC staining showing the infarct volume in brain sections. The representative images were placed from the anterior to the posterior portion of the brain. Scale bar = 10 mm. (**B**) The infarct volume was quantified via TTC staining. *N* = 5/group. (**C**) The Bederson score was assessed in rats on days 3 to 6 after MCAO. *N* = 10/group. (**D**) Quantitative analysis of the number of caspase-3-positive cells in the peri-infarct cerebral cortex. *N* = 5/group. (**E**) Cleaved caspase-3-positive cells (red) in the peri-infarct cerebral cortex. DAPI (blue) labeled the nuclei. The insets show photomicrographs of the SVZ at higher magnification. Arrows point to the caspase-3-positive cells. Scale bar = 45 μm. *N* = 5/group. * *p* < 0.05; *** *p* < 0.001 according to ANOVA followed by LSD post hoc analysis.

TTC staining of brain slices was performed 14 days after MCAO to determine the infarct volume. Rats treated with RADA16-I + CDNF after MCAO exhibited a significantly reduced infarct volume ((F(3, 12) = 4.378, *p* < 0.05); post hoc *p* < 0.05) compared to that of the control group (Figure 7A,B). Although the results failed to achieve statistical significance, rats treated with CDNF or RADA16-I alone showed a trend toward reduced infarct volume (post hoc *p* > 0.05).

Neurological deficits were assessed in rats on days 3 to 6 after MCAO. Rats in all groups showed no significant difference in Bederson score on day 3 or day 5 in the behavioral assays (all *p* > 0.05, Figure 7C). Rats treated with CDNF displayed a significant reduction in the Bederson score compared to the control group on days 4 ((F(3, 49) = 3.361, *p* = 0.026); post hoc *p* = 0.044) and 6 ((F(3, 49) = 3.306, *p* = 0.028); post hoc *p* = 0.023). However, no significant difference in Bederson score was obtained in rats treated with RADA16-I alone or RADA16-I + CDNF compared to that in the control group (all post hoc *p* > 0.05).

Immunostaining of cleaved caspase-3 was used to identify apoptosis in the ischemic penumbra area. Treatment after MCAO significantly reduced the number of apoptotic cells in the ischemic prenumbra area (F (3, 84) = 9.643; *p* < 0.0001, Figure 7D,E). Post hoc analysis revealed that the CDNF and RADA16-I + CDNF treatment groups had fewer cleaved caspase-3-positive cells than the control group (*p* < 0.001), although there was no significant difference between these two treatment groups (*p* > 0.05). The number of cleaved caspase-3-positive cells in the group treated with RADA16-I alone was not significantly different from that in the control group (*p* > 0.05). This result suggests that CDNF, not RADA16-I, reduces apoptosis in the ischemic penumbra area of MCAO rats.

Taken together, these results demonstrate that CDNF, not RADA16-I, has a neuroprotective effect in MCAO rats.

## 3. Discussion

In the present study, we investigated the effect of RADA16-I hydrogel and CDNF on the proliferation and differentiation of NSCs in vitro and on neurogenesis and neuroprotection in MCAO rats. First, CDNF promoted the proliferation of NSCs, while RADA16-I induced the neural differentiation of NSCs in vitro. Furthermore, RADA16-I and CDNF promoted the proliferation of NSCs and neuroblasts in the SVZ and neuronal differentiation in the ischemic penumbra area in vivo by activating the ERK1/2 and STAT3 pathways. Meanwhile, CDNF played a primary role in the migration of neuroblasts to the ischemic penumbra area. In addition, CDNF had a significant neuroprotective effect on brain ischemia-reperfusion injury. Our results indicate that RADA16-I hydrogel combined with CDNF activates endogenous NSCs for brain injury repair. This finding indicates the possibility of the potential application of RADA16-I hydrogel and CDNF in stroke therapy.

NSCs are self-renewing and multipotent cells that can differentiate into neurons, astrocytes and oligodendrocytes [20]. The self-renewal and differentiation of NSCs are precisely regulated by many factors. Results from a number of studies have indicated the effect of RADA16-I hydrogel or its modification on NSCs. For example, RADA16 and RADA16-SVVYGLR hydrogels were found to support rat NSC survival, although NSCs required more time to adapt to the peptide hydrogel environment [34]. RADA16 and its modification with laminin induced human NPC differentiation and maturation into dopaminergic neurons. RADA16-I with laminin induced the highest dopaminergic maturation of neurons [5]. The combination of RADA16-I and RADA16-I modified with IKVAV was the most beneficial in terms of cell proliferation, distribution, migration, neuronal lineage differentiation, and maturation of neonatal mouse NSCs [35]. The highest cell distribution, adhesion, and survival of mouse NSCs were observed in RADA16-I modified with PFSSTKT [36]. RADA16-I stimulated the differentiation and maturation of human ES-NPCs, and neurite outgrowth was greater [6]. Our data showed that RADA16-I had no proliferation effect in vitro, however, it did in vivo. The reason may be that RADA16-I trigger other niche cells in the SVZ to secret growth factors to promote the proliferation of NSCs and neuroblasts in vivo via an unknown mechanism. In agreement with these previous reports, we also found that RADA16-I promoted the neuronal lineage differentiation of rat NSCs. Studies have reported that MANF, another member of the CDNF/MANF family, promoted the differentiation and migration of neural progenitor cells in an OGD model [17], and MANF protein administration did not affect the growth of cells but triggered neuronal and glial differentiation in NSC cultures [17]. CDNF was able to reverse the adverse proliferation, differentiation, and apoptosis effects that normally affect rat NSCs in a hypoxic environment [19]. In the in vitro study, we found that CDNF only played roles in the proliferation of NSCs. Differences in the structure between CDNF and MANF or the concentration of CDNF in these studies may indicate the primary reason for these inconsistencies.

In the brain, NSCs of the SVZ give rise to NPCs that migrate through the rostral migratory stream to the olfactory bulb [37]. Ischemic injury activates endogenous NSCs in the SVZ, and the surviving neuroblasts migrate out of the SVZ into the ischemic area. However, this process is limited because the majority of NPCs either fail to reach the lesioned cortex or differentiate into glial cells due to a nonpermissive environment for neuronal differentiation [25,26]. Studies have reported that injected RADA16-I or RADA16-IKVAV supports survival and promotes proliferation of encapsulated NSCs after traumatic brain injury [8]. RADA 16-I microspheres encapsulated with human iPS-NPCs were injected into the mouse striata, which promoted neurite outgrowth and enhanced cell survival [9]. In a rat model of cortical stroke, injected MANF did not affect cell proliferation in the SVZ but promoted the migration of DCX-positive cells toward the corpus callosum and infarct boundary [37]. A study of MANF-deficient mice showed that MANF does not affect the self-renewal capacity or proliferation of NSCs; however, MANF plays a significant role in neurite growth in the process of neuronal differentiation and delayed neuronal migration [38]. In this study, we found that RADA16-I promoted the proliferation and neuronal differentiation of NSCs in the SVZ, while CDNF promoted the proliferation, migration, and neuronal differentiation of NSCs in MCAO rats. Although they have similar structures, CDNF and MANF may have different roles in neurogenesis.

As scaffold materials, hydrogels made of self-assembling peptides have been investigated in many in vitro and in vivo studies [3]. In vitro studies have shown that RADA16-I- and RADA16-I-modified hydrogels promote cell proliferation and migration and induce the neuronal differentiation of NSCs [5,35,36,39]. Furthermore, in vivo data are also promising for the translation of hydrogels with NSCs into a brain injury model [7,8,9]. However, the molecular mechanism of the role of RADA16-I- and RADA16-I-modified hydrogels on NSCs remains unknown.

To date, the signaling pathways activated by CDNF are still largely unclear because the putative CDNF receptors are unknown. Published data from our lab showed that CDNF has neuroprotective effects on cerebral ischemia through ER stress pathways [16]. For neurogenesis, there are no published data related to CDNF; however, MANF promotes the differentiation and migration of NSCs by activating the STAT3 and ERK1/2 pathways [17]. STAT3 is involved in neuronal differentiation, and serine-phosphorylated STAT3 is involved in neurite outgrowth induced by nerve growth factor (NGF) [40,41]. The ERK1/2 pathway has been suggested to be involved in Ser727 phosphorylation of STAT3 [42]. Many studies have shown that the ERK1/2 pathway regulates the differentiation of NSCs through many mechanisms and is implicated in cell migration induced by growth factors [43,44,45,46]. In this study, we found that STAT3 phosphorylation at Ser727 is induced following CDNF treatment, while RADA16-I hydrogel combined with CDNF induced the phosphorylation of ERK1/2. These findings suggested that CDNF promotes neuronal differentiation via activation of the STAT3 pathway. This is consistent with the effect of MANF on NSCs [17]. The reason CDNF-only treatment did not activate the ERK1/2 pathway may be the different phosphorylation peak times between the ERK1/2 and STAT3 pathways. Further studies will be required to determine the putative CDNF receptors on NSCs and the crosslink between the ERK1/2 and STAT3 signaling pathways in neurogenesis.

As a novel NTF, the neuroprotective effect of CDNF has been previously reported [13,14,15,16]. In addition to its neurotrophic activities in dopaminergic neurons, the neuroprotective effect of CDNF was observed in MCAO rats [16]. Both CDNF and MANF reduced the volume of infarction, attenuated apoptotic cells and improved motor function in ischemic brain injury [16,47]. Consistent with these studies, our data are in general agreement with the findings that CDNF exerts neuroprotective effects on cerebral ischemia, while RADA16-I had no obvious neuroprotective effect. The results from our present study suggest that RADA16-I may have no direct neuroprotective effect; however, it needs more time to exert this effect by working on neurogenesis. Delaying the investigated days may have adverse results.

In conclusion, we demonstrated that RADA16-I hydrogel and CDNF promote neurogenesis and neuroprotection in MCAO rats. In addition, we tested the effect of RADA16-I hydrogel and CDNF on the proliferation and differentiation of NSCs in vitro. CDNF promoted the proliferation of NSCs, while RADA16-I induced the neural differentiation of NSCs in vitro. More interestingly, both RADA16-I and CDNF promoted the proliferation, migration, and differentiation of NSCs by activating the ERK1/2 and STAT3 pathways in vivo, and CDNF exerted an obvious neuroprotective effect on brain ischemia-reperfusion injury. Collectively, these findings provide new information regarding the application of the scaffold material RADA16-I hydrogel and the neurotrophic factor CDNF in neural tissue engineering and suggest that RADA16-I hydrogel and CDNF microinjection may represent a novel therapeutic strategy for the treatment of stroke.

## 4. Materials and Methods

### 4.1. Experimental Materials

In this study, healthy 2-month-old adult male Sprague–Dawley rats (280–350 g) were obtained from Vital River Laboratories (Beijing, China). The temperature of the feeding environment was controlled at 22 ± 2 °C, and the rats were housed on a 12-h light/dark cycle. All procedures were approved by the Ethics Committee on Animal Experiments of School of Basic Medical Sciences of Shandong University. RADA16-I (AcN-RADARADARADARADA-CNH2) was synthetized by Shanghai Botai Biotechnology Co. Ltd. (Shanghai, China). RADA16-I (1%) was dissolved in sterile distilled water and stored at −20 °C. Recombinant human CDNF protein (R&D Systems Inc., Minneapolis, MN, USA) was dissolved in PBS.

### 4.2. NSC Culture

As previously described [48], NSCs were obtained from Sprague–Dawley rats on gestational day 14. The cortical neuroepithelium was dissected and digested with 0.2% papain (Sigma, St. Louis, MO, USA) at 37 °C for 30 min. The tissue was dissociated into a single cell suspension, and cells were plated at a density of 1 × 10^5^ cells/mL in DMEM/F12 medium supplemented with 2% B27 (both from Invitrogen, Carlsbad, CA, USA). EGF and bFGF (20 ng/mL, both from Peprotech, Rocky Hill, NJ, USA) were added to the medium. Cells were passaged approximately every 7 days.

### 4.3. Immunocytochemistry

For the differentiation of NSCs, cells were plated on poly-d-lysine-coated cover slips in DMEM/F12 medium with 1% FBS ( Invitrogen, Carlsbad, CA, USA) instead of growth factors for 7 days. Cells were washed in PBS three times and fixed in 4% formaldehyde for 10 min. Triton X-100 (0.2%) was used for permeabilization for 10 min at room temperature. Then, the cells were blocked in 1% BSA (Solarbio, Beijing, China) in PBS for 20 min at room temperature. Next, the cells were incubated with primary antibodies (rabbit polyclonal anti-glial fibrillary acidic protein (GFAP) 1:1000, Abcam, Cambridge, MA, USA, and mouse monoclonal anti-neuronal class III β-Tubulin (Tuj1) 1:500, Abcam, Cambridge, MA, USA) overnight at 4 °C. After washing with PBS, the cells were incubated with secondary antibodies (Alexa Fluor 488-conjugated IgG 1:1000 and Alexa Fluor 594-conjugated IgG 1:1000, Invitrogen, Carlsbad, CA, USA) in PBS for 40 min at 37 °C. Images were captured using a Nikon 80i light microscope equipped with a CCD camera.

### 4.4. MCAO Model

First, rats were anesthetized with 10% chloral hydrate via intraperitoneal injection (4 mL/kg body weight). The right common carotid artery (CCA), external carotid artery (ECA), and internal carotid artery (ICA) were isolated. A nylon filament (0.38 mm in diameter) with an expanded tip was gently advanced from the CCA into the lumen of the ICA to block the origin of the middle cerebral artery (MCA). The right MCA was occluded with the filament for 2 h, and then 24 h of reperfusion was performed by withdrawing the filament. A right neck incision was also made to expose the arteries in the sham group. However, nylon thread was not inserted into the internal carotid artery. The rats were returned to their home cages following recovery from anesthesia.

### 4.5. Intracerebroventricular Microinjection

After MCAO for three days, rats were subjected to intracerebroventricular microinjection of RADA16-I, CDNF, RADA16-I+CDNF, or vehicle (*n* = 20 for each group). Each rat was placed into a stereotaxic frame after anesthesia with 10% chloral hydrate. The head was further stabilized in a customized head mold. The right lateral ventricle was targeted at the following coordinates from bregma: −0.9 mm anterior, ±1.5 mm lateral and −3.6 mm dorsal. Two microliters of RADA16-I (10 µg), CDNF (6 µg), RADA16-I (10 µg) combined with CDNF (6 µg), or sterile water was injected at a rate of 1 µL/min. The needle was retained in place for 5 min after following the injection. 

### 4.6. BrdU Labeling

After intracerebroventricular microinjection, rats were intraperitoneally injected with 50 mg/kg BrdU (Sigma, St. Louis, MO, USA) once a day for 7 consecutive days. To label cell proliferation and migration, rats were sacrificed 11 days after MCAO. The rats were transcardially perfused with normal saline followed by 4% paraformaldehyde. The brains were then removed and fixed in 4% paraformaldehyde.

### 4.7. Immunohistochemistry

The rat brains were cut into serial sagittal or coronal (40 µm) sections using a cryostat and stored at −80 °C. According to the protocol for immunofluorescence staining, sections were incubated overnight with primary antibody at 4 °C. The primary antibodies used were sheep monoclonal anti-BrdU (1:500; Cell Signaling Technology, Danvers, MA, USA), rabbit monoclonal anti-doublecortin (DCX, 1:500; Cell Signaling Technology, Danvers, MA, USA), mouse monoclonal anti-nestin (1:500; Millipore, Billerica, MA, USA), mouse monoclonal anti-Tuj1 (1:500; Abcam, Cambridge, MA, USA), rabbit polyclonal anti-GFAP (1:1000; Millipore, Billerica, MA, USA), mouse monoclonal anti-NeuN (1:1000; Millipore, Billerica, MA, USA), and rabbit monoclonal anti-cleaved caspase-3 (1:500; Cell Signaling Technology, Danvers, MA, USA). The secondary antibodies used were Alexa Fluor 488-conjugated IgG (1:1000; Invitrogen, Carlsbad, CA, USA) and Alexa Fluor 594-conjugated IgG (1:1000; Invitrogen, Carlsbad, CA, USA). The nuclei of the cells were counterstained with 4,6-diamidino-2-phenylindole (DAPI).

Every sixth section was collected for staining. To quantify the staining, images were digitally captured using a Nikon 80i light microscope equipped with a CCD camera under a 20× objective. Blinded cell counting was applied by NIH Image J. The number of different positive cells was counted in the different brain area per rat in each group. 

### 4.8. Evaluation of Infarct Volume

Following the published protocol [49], the brains of rats were carefully removed and cut into 2.0 mm thick coronal sections after sacrifice. Fresh brain slices were incubated in 2% triphenyltetrazolium chloride (TTC, Sigma, St. Louis, MO, USA) in normal saline for 30 min at 37 °C. Then, the sections were transferred to a 4% paraformaldehyde solution for fixation. Digital images were acquired after staining. The area of infarction in each slice was measured with NIH ImageJ software, version 1.46 (Bethesda, MA, USA). The infarct areas of each section were obtained as the average of the sum of two sides. The volume of infarction for each animal was calculated by multiplying the average slice thickness (2 mm) by the sum of the infarct areas in all brain slices. The results are represented as a percentage of the total volume.

### 4.9. Examination of Neurological Deficits

Behavioral assays were performed as previously described [49]. Briefly, the rats were examined on days three to six after MCAO. While suspended 20–30 cm above the testing table, the rats were scored according to the following criteria: 0, rat extends both forelimbs straight; 1, rat keeps one forelimb to the breast and extends the other forelimb straight; 2, rat exhibits decreased resistance to a lateral push without circling; 3, rat exhibits decreased resistance to a lateral push with circling; and 4, rat unable to spontaneously walk and loses consciousness. Rats scoring 0 or 4 were not used in the evaluation of this experiment.

### 4.10. Western Blotting

Eleven days after intracerebroventricular microinjection, the tissues in the penumbra around the infarct area were extracted and lysed in lysis buffer containing 1% protease inhibitor and 1% phosphatase inhibitor. Total protein was separated using SDS–PAGE and transferred to a nitrocellulose membrane. The membrane was blocked and incubated with an appropriate primary antibody and secondary antibody. The primary antibodies were mouse anti-tubulin (1:1000, Cell Signaling Technology, Danvers, MA, USA), anti-p-ERK (1:300, Wanleibio, Shen Yang, China), anti-p-STAT3 (1:1000, Wanleibio, Shen Yang, China), anti-ERK (1:2000, Cell Signaling Technology, Danvers, MA, USA) and anti-STAT3 (1:400, Wanleibio, Shen Yang, China). Horseradish peroxidase (HRP)-conjugated secondary antibodies (1:5000; Millipore, Billerica, MA, USA) were utilized. All immunoblotting experiments were repeated at least three times.

### 4.11. Statistical Analysis

All data are expressed as the means ± standard error of the mean (SEM). Differences between groups were analyzed via one-way ANOVA followed by LSD post hoc analysis. Statistical significance was set at *p* < 0.05.

## Supplementary Materials

The following supporting information can be downloaded at: https://www.mdpi.com/article/10.3390/ijms23031436/s1.

## Abbreviations

CNS	central nervous system
DAPI	4,6-diamidino-2-phenylindole dihydrochloride hydrate
CDNF	cerebral dopamine neurotrophic factor
NSCs	neural stem cells
LV	lateral ventricle
MCAO	middle cerebral artery occlusion
NTFs	neurotrophic factors
NGF	nerve growth factor
BDNF	brain-derived neurotrophic factor
GDNF	glial cell line-derived neurotrophic factor
OGD	oxygen-glucose deprivation
SVZ	subventricular zone
SGZ	subgranular zone
DG	dentate gyrus
MANF	mesencephalic astrocyte-derived neurotrophic factor
GFAP	glial fibrillary acidic protein
CCA	common carotid artery
ECA	external carotid artery
ICA	internal carotid artery
MCA	middle cerebral artery
DCX	doublecortin
TTC	triphenyltetrazolium chloride
SEM	standard error of the mean
HRP	horseradish peroxidase
