# Supplementary figures and images for: The Effect of RADA16-I and CDNF on Neurogenesis and Neuroprotection in Brain Ischemia-Reperfusion Injury
Source: Int J Mol Sci. 2022 Jan 27;23(3):1436. doi: 10.3390/ijms23031436 (PMC8836142; doi:10.3390/ijms23031436)

A

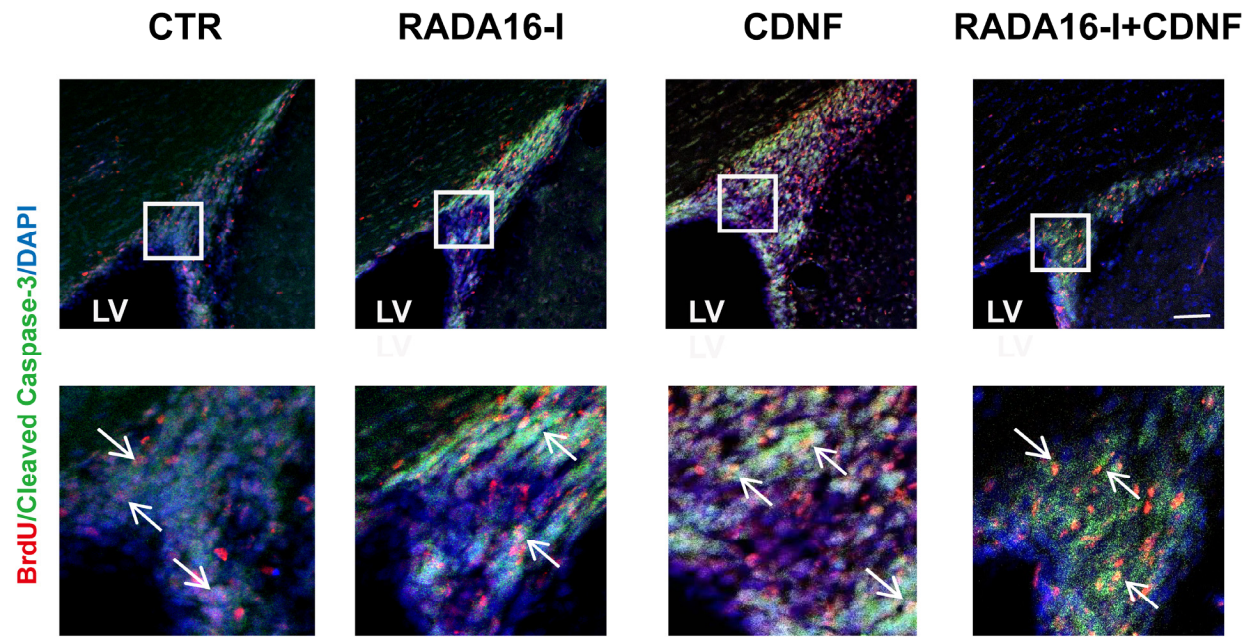

B

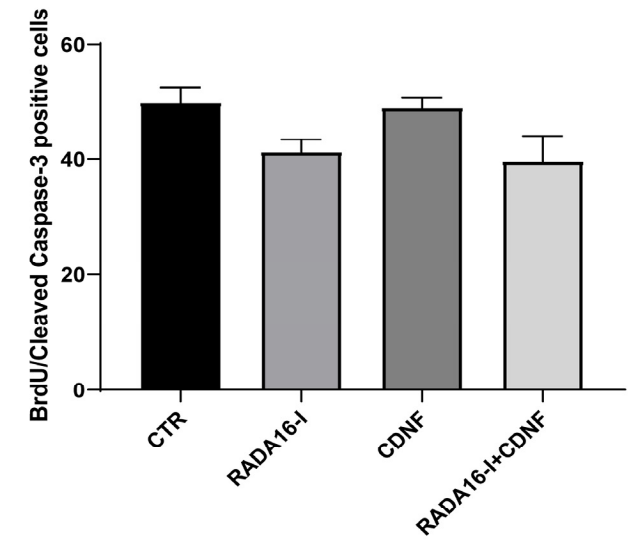

Supplement: Supplementary file 1 [file ijms-23-01436-s001.zip › ijms-1541809-supplementary.pdf]
